# IN THE PRESENT AND IN THE FUTURE: HOW TIME PERCEPTION AND TIME ORIENTATION SHAPE WELL-BEING ACROSS ADULT LIFE

**DOI:** 10.1093/geroni/igad104.1817

**Published:** 2023-12-21

**Authors:** Yoonseok Choi, Li Chu, Laura Carstensen

## Abstract

Individuals are able to maintain or even improve their wellbeing well into older adulthood. Socioemotional selectivity theory describes such age-related improvement in terms of motivational shifts toward emotionally meaningful goals with shrinking future time horizons. However, the association between future time perceptions and well-being is in need of being further clarified. In this symposium, four presentations will shed light on three different aspects of future time perceptions — time savoring, future preoccupation, thinking about the future — and their associations with wellbeing. Growney et al will present a study that utilizes a day reconstruction method to examine if time savoring is associated with psychosocial wellbeing and helping behaviors in workplaces. Pauly et al will discuss the potential benefits of time savoring for how solitude is experienced, by examining the buffering role of time savoring in depressive mood and somatic symptoms during solitude. Chu et al offers an alternative conceptualization of future time horizons, future preoccupation, capturing the worries and fixation on future preparation, and discusses its role in wellbeing across the life span. Choi et al discuss the role of thinking about the future for age-related differences in everyday experiences of happiness of varying arousal levels. The symposium will end with a discussion by Laura Carstensen integrating these findings into research on future time perspective and wellbeing across the life span. By elucidating more specific time-related experiences, our findings contribute to a more comprehensive understanding of how age-related changes in future time perspectives shape wellbeing trajectories across the life span.

